# Supplementary material for: Age-Stratified Analysis of First-Line Chemoimmunotherapy for Extensive-Stage Small Cell Lung Cancer: Real-World Evidence from a Multicenter Retrospective Study
Source: Cancers (Basel). 2023 Feb 28;15(5):1543. doi: 10.3390/cancers15051543 (PMC10001399; doi:10.3390/cancers15051543)
Supplement: Supplementary file 1 [file cancers-15-01543-s001.zip › cancers-2226932-supplementary.pdf]

Supplementary Table S1. Response to treatment

	**Total: n=155**	**Non-elderly patients (< 75 years): n= 98**	**Elderly patients (≥ 75 years): n=57**	**p-value**
Response				
Complete response	2 (1.3%)	2 (2.0%)	0 (0%)	
Partial response	124 (80.0%)	77 (78.6%)	47 (82.5%)	
Stable disease	20 (12.9%)	14 (14.3%)	6 (10.5%)	
Progressive disease	5 (3.2%)	4 (4.1%)	1 (1.8%)	
Not evaluable	4 (2.6%)	1 (1.0%)	3 (5.3%)	
Objective response rate (95% CI)	81.3%	80.6%	82.5	0.83
Disease control rate (95% CI)	94.2%	94.9%	93.0	0.73

Supplementary Table S2. Treatment-related adverse events

	**Total: n=155**		**Non-elderly patients (< 75 years): n= 98**		**Elderly patients (≥ 75 years): n=57**	
**Adverse events**	**Any grade (%)**	**Grade ≥3 (%)**	**Any grade (%)**	**Grade ≥3 (%)**	**Any grade (%)**	**Grade ≥3 (%)**
Leading to any treatment withdrawal	12 (7.7%)	6 (3.9%)	6 (6.1%)	3 (3.1%)	6 (10.5%)	3 (5.3%)
Neutropenia	114 (73.5%)	109 (70.3%)	68 (69.4%)	65 (66.3%)	46 (80.7%)	44 (77.2%)
Anemia	41 (26.5%)	10 (6.5%)	31 (31.6%)	7 (7.1%)	10 (17.5%)	3 (5.3%)
Thrombocytopenia	47 (30.3%)	19 (12.3%)	32 (32.7%)	12 (12.2%)	15 (26.3%)	7 (12.3%)
Febrile neutropenia	16 (10.3%)	16 (10.3%)	11 (11.2%)	11 (11.2%)	5 (8.8%)	5 (8.8%)
Sepsis	1 (0.6%)	1 (0.6%)	0	0	1 (1.8%)	1 (1.8%)
Nausea	10 (6.5%)	1 (0.6%)	9 (9.2%)	1 (1.0%)	1 (1.8%)	0
Anorexia	6 (3.9%)	0	5 (5.1%)	0	1 (1.8%)	0
Hiccup	3 (1.9%)	0	2 (2.0%)	0	1 (1.8%)	0
Constipation	9 (5.8%)	1 (0.6%)	7 (7.1%)	1 (1.0%)	2 (3.5%)	0
Mucositis oral	1 (0.6%)	0	1 (1.0%)	0	1 (1.8%)	0
Lung infection	2 (1.3%)	2 (1.3%)	0	0	2 (3.5%)	2 (3.5%)

Fatigue	10 (6.5%)	2 (1.3%)	8 (8.2%)	0	2 (3.5%)	2 (3.5%)
Insomnia	1 (0.6%)	0	0	0	1 (1.8%)	0
Alopecia	6 (3.9%)	0	4 (4.1%)	0	2 (3.5%)	0
Aspartate aminotransferase increased	4 (2.6%)	0	2 (2.0%)	0	2 (3.5%)	0
Creatinine increased	2 (1.3%)	0	2 (2.0%)	0	0	0
Hyponatremia	7 (4.5%)	5 (3.2%)	6 (6.1%)	5 (5.1%)	1 (1.8%)	0
Hyperkalemia	1 (0.6%)	0	1 (1.0%)	0	0	0
Hypokalemia	1 (0.6%)	1 (0.6%)	1 (1.0%)	1 (1.0%)	0	0
Hypomagnesemia	1 (0.6%)	0	1 (1.0%)	0	0	0
Stroke	1 (0.6%)	0	0	0	1 (1.8%)	0
Tumor lysis syndrome	1 (0.6%)	1 (0.6%)	0	0	1 (1.8%)	1 (1.8%)
Tinnitus	1 (0.6%)	0	1 (1.0%)	0	0	0
Peripheral sensory neuropathy	2 (1.3%)	0	2 (2.0%)	0	0	0
Urinary incontinence	1 (0.6%)	0	1 (1.0%)	0	0	0
Palmar-plantar	1 (0.6%)	0	1 (1.0%)	0	0	0

erythrodysesthesia syndrome						
Immune-related adverse events						
Rash maculo-papular	8 (5.2%)	0	5 (5.1%)	0	3 (5.3%)	0
Hyperthyroidism	5 (3.2%)	2 (1.3%)	5 (5.1%)	2 (2.0%)	0	0
Hypothyroidism	4 (2.6%)	0	3 (3.1%)	0	1 (1.8%)	0
Adrenal insufficiency	2 (1.3%)	0	0	0	2 (3.5%)	0
Pneumonitis	7 (4.5%)	3 (1.9%)	4 (4.1%)	2 (2.0%)	3 (5.3%)	1 (1.8%)
Colitis	2 (1.3%)	0	1 (1.0%)	0	1 (1.8%)	0
Myasthenia gravis	1 (0.6%)	1 (0.6%)	1 (1.0%)	1 (1.0%)	0	0
Encephalopathy	1 (0.6%)	1 (0.6%)	0	0	1 (1.8%)	1 (1.8%)
Vasculitis	1 (0.6%)	1 (0.6%)	1 (1.0%)	1 (1.0%)	0	0
